# Performance of severity indices for admission and mortality of trauma patients in the intensive care unit: a retrospective cohort study

**DOI:** 10.1186/s40001-023-01532-6

**Published:** 2023-12-04

**Authors:** Tatiane Gonçalves Gomes de Novais do Rio, Lilia de Souza Nogueira, Fernanda Rodrigues Lima, Carolina Cassiano, Diogo de Freitas Valeiro Garcia

**Affiliations:** 1 Nursing Department, Samaritano Higienópolis Hospital, São Paulo, Brazil; 2 Medical-Surgical Nursing Department, School of Nursing, University of São Paulo, São Paulo, Brazil; 3 School of Nursing, University of São Paulo, São Paulo, Brazil; 4 Nursing Department, Faculdade de Ciências Médicas da Santa Casa de São Paulo, São Paulo, Brazil; 5 Nove de Julho University, São Paulo, Brazil

**Keywords:** Trauma, Trauma severity indices, Intensive care units, Mortality, ROC Curve

## Abstract

**Background:**

Little is known about the performance of severity indices for indicating intensive care and predicting mortality in the Intensive Care Unit (ICU) of trauma patients. This study aimed to compare the performance of severity indices to predict trauma patients’ ICU admission and mortality.

**Methods:**

A retrospective cohort study which analyzed the electronic medical records of trauma patients aged ≥ 18 years, treated at a hospital in Brazil, between 2014 and 2017. Physiological [Revised Trauma Score (RTS), New Trauma Score (NTS) and modified Rapid Emergency Medicine Score (mREMS)], anatomical [Injury Severity Score (ISS) and New Injury Severity Score (NISS)] and mixed indices [Trauma and Injury Severity Score (TRISS), New Trauma and Injury Severity Score (NTRISS), Base-deficit Injury Severity Score (BISS) and Base-deficit and New Injury Severity Score (BNISS)] were compared in analyzing the outcomes (ICU admission and mortality) using the Area Under the Receiver Operating Characteristics Curves (AUC–ROC).

**Results:**

From the 747 trauma patients analyzed (52.5% female; mean age 51.5 years; 36.1% falls), 106 (14.2%) were admitted to the ICU and 6 (0.8%) died in the unit. The ISS (AUC 0.919) and NISS (AUC 0.916) had better predictive capacity for ICU admission of trauma patients. The NISS (AUC 0.949), TRISS (AUC 0.909), NTRISS (AUC 0.967), BISS (AUC 0.902) and BNISS (AUC 0.976) showed excellent performance in predicting ICU mortality.

**Conclusions:**

Anatomical indices showed excellent predictive ability for admission of trauma patients to the ICU. The NISS and the mixed indices had the best performances regarding mortality in the ICU.

## Background

Trauma is responsible for significant mortality and rates of Intensive Care Unit (ICU) admissions [1, 2]. In this context, the trauma registry is a fundamental part of quality programs and seeks to systematically store data that reflect the real impact of trauma and injuries on the clinical outcome of victims [3].

Trauma severity indices are among the data which compose the trauma registry. These constitute scoring systems that assess physiological and/or biochemical changes and/or the severity of traumatic injuries, enabling to identify the severity of the trauma [4, 5].

The Revised Trauma Score (RTS) stands out among the physiological severity indices [6], with an improved version called the New Trauma Score (NTS) [7], as well as the modified Rapid Emergency Medicine Score (mREMS) [8]. Furthermore, the Injury Severity Score (ISS) and the New Injury Severity Score (NISS) are the most applied anatomical indices in practice [9, 10]. The combination of physiological and anatomical parameters resulted in creating a mixed index called the Trauma and Injury Severity Score (TRISS) [11], and its improved version called the New Trauma and Injury Severity Score (NTRISS) [12]. The Base-deficit Injury Severity Score (BISS) [5, 13] and the Base-deficit and New Injury Severity Score (BNISS) [5] are also classified as mixed indices, and consider the base excess (BE) marker in assessing trauma severity.

It is noteworthy that the performance of trauma indices for predicting the probability of in-hospital survival or mortality is frequently addressed in different studies [5, 7, 14–16]; however, the analysis of the predictive capacity of these indices for admission and mortality in the ICU is scarce. Results show that the ISS and NISS perform well in identifying severe trauma patients who need intensive care [17, 18] and the TRISS and the mREMS are highlighted as predictors of death in the ICU of this population [19, 20].

Considering the diversity of severity indices available in the literature and that little is known about their ability to predict admission and mortality of trauma patients in the ICU, the relevance of this study which seeks to find an index that safely enables early identification of patients who really need intensive care in relation to those for whom admission to a ward would be sufficient is emphasized, in addition to victims of severe trauma who are more likely to die in the critical unit.

## Materials and methods

This is a retrospective cohort study aimed to compare the performance of severity indices to predict trauma patients’ ICU admission and mortality. Carried out in a Samaritano Hospital Trauma Center located in São Paulo, Brazil. The sample consisted of trauma patients aged 18 years or over, admitted between January 1, 2014 and December 31, 2017 at the institution within 24 h after a traumatic event. Patients who arrived in cardiac arrest without resuscitation success in the emergency room and victims of burns, drowning, poisoning, asphyxiation or suffocation were excluded from the study.

The dependent variables of the study were admission and mortality in the ICU. The independent variables included physiological (RTS, NTS and mREMS), anatomical (ISS and NISS) and mixed (TRISS, NTRISS, BISS and BNISS) indices.

The RTS assigns points (from zero to 4) to three physiological parameters of the trauma patient: Systolic Blood Pressure (SBP), Respiratory Rate (RR) and Glasgow Coma Scale (GCS) score. In the hospital context, the values of the RTS variables (SBP, RR and GCS) are multiplied by their respective weights, which can range from zero to 7.8408 (the lower the value, the greater the patient’s severity) [6]. The NTS is a modification of the RTS, and considers the integer corresponding to the GCS score for its calculation, revises the ranges of SBP values proposed by the RTS and replaces the RR by variations in peripheral oxygen saturation (SpO_2_), and its final score can range from 1.202 (most severe) to 10.685 (less severe) [7]. The most recent physiological index (the mREMS) is obtained by the sum of the scores attributed to the variables SBP, Heart Rate (HR), RR, SpO_2_, GCS and age of the trauma patient, ranging from zero to 26, which is the maximum score that reflects higher probability of death [8].

To calculate the ISS, it is necessary to identify all anatomical injuries diagnosed in trauma victims and their respective scores obtained on the Abbreviated Injury Scale (AIS), which is an instrument that provides an identifier composed of seven numbers for each injury description, with the last digit reflecting the AIS severity score, and ranges from one (less severe) to six points (maximum severity) [21]. The ISS considers six body regions (head and neck, face, chest, abdomen and pelvic contents, extremities or pelvic girdle and external surface) and is calculated by summing the square of the highest AIS of three distinct body regions [9]. The NISS was created to mitigate the weaknesses of the ISS, which underestimates the severity of trauma with multiple severe injuries occurring in the same body region. The three most serious injuries identified by the AIS are also considered to calculate the NISS, regardless of the affected body region [10]. The ISS and NISS can range from 1 to 75 points, and the higher the value, the greater the trauma severity [9, 10].

The RTS value of the patient’s admission to the emergency service, the ISS, the victim’s age and the type of trauma (blunt or penetrating) are considered to calculate the TRISS, enabling to identify the trauma victim’s survival probability through regression logistics [11]. The TRISS also had its update with the emergence of the NTRISS. The NTRISS calculation is based on the same formula as TRISS with the replacement of the ISS value by NISS [12].

The BISS calculation is also based on a mathematical logistic regression formula and provides the survival probability of the trauma patient through an analysis of age, ISS and BE delta (ΔBE), replacing the RTS considered in TRISS [5, 13]. Finally, the BNISS [5] replaces ISS with NISS in the BISS formula.

Data for this study were collected by analyzing electronic medical records of trauma patients. Physiological parameters were retrieved from the emergency room care records and considered the values recorded at the time the patient was admitted to the institution. The BE value was identified through arterial blood gas collected upon the patient’s admission to the ICU.

All traumatic injuries registered in the patient’s medical record during their stay in the institution and diagnosed through physical examination, surgical interventions and imaging tests were considered. The AIS code was identified for each anatomical lesion through the AIS 2008 update 2015 manual [21]. The indices were calculated by two researchers in the trauma area, and a third researcher was consulted if there was disagreement between them, with the majority opinion prevailing.

Receiver Operating Characteristic (ROC) curves were constructed to assess the performance of trauma indices, obtaining measurements of area under the curve (AUC), confidence interval, sensitivity, specificity, positive predictive value (PPV), negative predictive value (NPV) and accuracy. The Youden’s index was applied to identify the best cutoff point for each index, while considering the best sensitivity and specificity in relation to the variable addressed. AUC values greater than 0.900 were considered excellent. The comparison between pairs of indices that presented AUC greater than 0.900 was performed by DeLong tests (comparing indices that present results with the same direction, for example, TRISS and BISS) and Hanley–McNeil (comparing indices that present results with opposite directions, i.e. the ISS and TRISS). The significance level adopted in all analyzes was 5%.

This study was approved by the Research Ethics Committee of the Samaritano Hospital (opinion number 2,793,810) that waived the Informed Consent Form to the participants as this is a study with data collection from secondary sources (medical records).

## Results

A total of 747 trauma victims were included in the study (52.5% female; mean age 51.5 years). The blunt trauma (*n* = 668; 89.4%) and falls (*n* = 270; 36.1%) prevailed. Data in Table 1 show low severity of trauma patients in the sample, as evidenced by means and medians of the indices close to normal values.

**Table 1:** Physiological, anatomical and mixed severity indices

Trauma severity indices	Mean (standard deviation)	Median	Minimum	Maximum
RTS	7.8 (0.3)	7.8	4.1	7.8
NTS	10.5 (0.5)	10.7	4.4	10.7
mREMS	2.1 (2.3)	1	0	20
ISS	3.4 (5.5)	1	0	45
NISS	4.3 (7.1)	1	0	66
TRISS	98.6 (3.0)	99.4	52.6	99.7
NTRISS	98.2 (4.9)	99.4	13.1	99.7

**Fig. 1:**
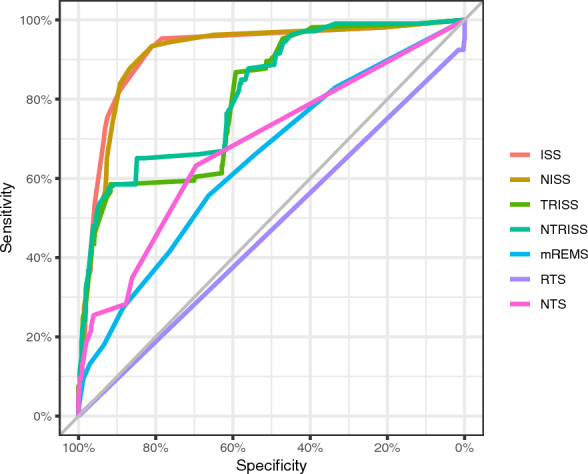
ROC curves of the performance of trauma severity indices in predicting patient admission to the ICU

A total of 106 patients (14.2%) were admitted to the ICU. Data in Fig. 1 and Table 2 show that the ISS and NISS had better predictive capacity for patient admission to the ICU with AUC values greater than 0.900 (ISS AUC 0.919; NISS AUC 0.916) compared to the other indices (RTS, NTS, mREMS, TRISS and NTRISS), in addition to satisfactory (above 80%) sensitivity, specificity, NPV and accuracy results. The cutoff point for the NISS (4.5) was higher than the ISS (3.5). The comparison of AUC values for ISS and NISS showed similarity in performance between the indices (*p* = 0.380), showing that both ISS and NISS are good predictors of patients who need intensive care.

**Table 2:** Comparison of the performance of trauma severity indices in predicting patient admission to the ICU

Trauma severity indices	AUC	95%CI	Cutoff point	Sens (%)	Spec (%)	PPV (%)	NPV (%)	Accuracy (%)
RTS	0.470	0.444–0.496	7.0	7.5	99.7	80.0	86.7	86.6
NTS	0.683	0.629–0.737	10.5	63.2	69.6	25.6	92.0	68.7
mREMS	0.645	0.587–0.702	2.5	55.7	66.3	21.5	90.0	64.8
ISS	0.919	0.880–0.949	3.5	93.4	81.0	44.8	98.7	82.7
NISS	0.916	0.885–0.946	4.5	87.7	86.7	52.3	97.7	86.9
TRISS	0.803	0.759–0.848	98.3	58.5	91.7	53.9	93.0	87.0
NTRISS	0.818	0.774–0.862	98.3	65.1	84.9	41.6	93.6	82.1

*RTS* Revised Trauma Score,* NTS* New Trauma Score,* mREMS* modified Rapid Emergency Medicine Score,* ISS* Injury Severity Score,* NISS* New Injury Severity Score,* TRISS* Trauma and Injury Severity Score,* NTRISS* New Trauma and Injury Severity Score, *AUC* Area Under the Curve,* 95% CI* 95% confidence interval,* Sens* Sensitivity; Spec, Specificity,* PPV* Positive Predictive Value,* NPV* Negative Predictive Value

The BNISS had a lower mean (78.8%) and median (83.9%) survival probability than the BISS (82.9% and 87.2%, respectively) among patients admitted to the ICU (*n* = 106). A total of 6 patients (0.8%) died in the ICU.

**Fig. 2:**
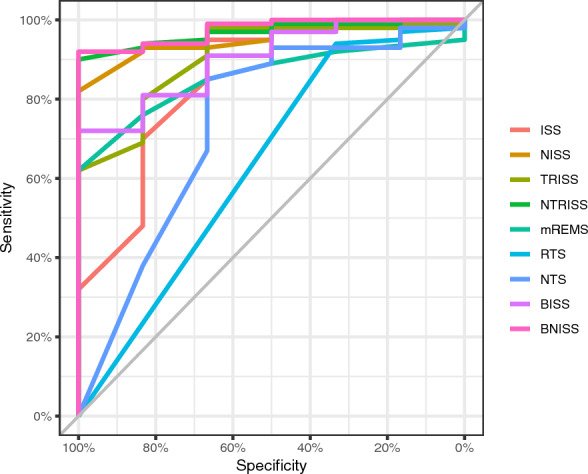
ROC curves of the performance of trauma severity indices in predicting ICU patient mortality

Data in Fig. 2 and Table 3 show that NISS, TRISS, NTRISS, BISS and BNISS showed excellent performance in predicting mortality in the ICU of these patients with AUC values above 0.900. With the exception of TRISS, these indices portrayed sensitivity and NPV of 100.0%. On the other hand, TRISS was the one with the highest accuracy (96.2%) and PPV (66.7%).

**Table 3:** Comparison of the performance of trauma severity indices in predicting ICU patient mortality

Trauma severity indices	AUC	95% CI	Cutoff point	Sens (%)	Spec (%)	PPV (%)	NPV (%)	Accuracy (%)
ISS	0.845	0.657–1.000	23.5	66.7	95.0	44.4	97.9	93.4
NISS	0.949	0.897–1.000	21.0	100.0	82.0	25.0	100.0	83.0
TRISS	0.909	0.804–1.000	81.7	66.7	98.0	66.7	98.0	96.2
NTRISS	0.967	0.930–1.000	91.6	100.0	90.0	37.5	100.0	90.6
mREMS	0.858	0.765–0.952	3.5	100.0	62.0	13.6	100.0	64.2
RTS	0.635	0.431–0.839	7.4	33.3	94.0	25.0	95.9	90.6
NTS	0.734	0.479–0.990	9.6	66.7	85.0	21.1	97.7	84.0
BISS	0.902	0.805–0.999	78.3	100.0	72.0	17.7	100.0	73.6
BNISS	0.976	0.943–1.000	57.4	100.0	92.0	42.9	100.0	92.5

*RTS* Revised Trauma Score,* NTS New* Trauma Score,* mREMS* modified Rapid Emergency Medicine Score,* ISS* Injury Severity Score,* NISS* New Injury Severity Score,* TRISS* Trauma and Injury Severity Score,* NTRISS* New Trauma and Injury Severity Score,* BISS* Base-deficit Injury Severity Score,* BNISS* Base-deficit and New Injury Severity Score, * AUC* Area Under the Curve,* 95% CI* 95% confidence interval,* Sens* Sensitivity,* Spec* Specificity,* PPV* Positive Predictive Value,* NPV* Negative Predictive Value

There was no significant difference (*p* > 0.005) in AUC values (Table 4), showing that any of the NISS, TRISS, NTRISS, BISS and BNISS indices are a good predictor of trauma patient mortality in the ICU.

**Table 4:** Comparison of AUC values of NISS, TRISS, NTRISS, BISS and BNISS indices for predicting ICU patient mortality

Compared indices	*p* value
NISS x TRISS	0.335*
NISS x NTRISS	0.335*
NISS x BISS	0.314*
NISS x BNISS	0.214*
TRISS x NTRISS	0.197**
TRISS x BISS	0.932**
TRISS x BNISS	0.244**
NTRISS x BISS	0.196**
NTRISS x BNISS	0.695**
BISS x BNISS	0.174**

*NISS* New Injury Severity Score,* TRISS* Trauma and Injury Severity Score,* NTRISS* New Trauma and Injury Severity Score,* BISS* Base-deficit Injury Severity Score,* BNISS* Base-deficit and New Injury Severity Score

^*Hanley–McNeil test^

^**DeLong test^

## Discussion

The results of this study made it possible to identify that the ISS or NISS indices can help in the decision-making process regarding the referral of trauma patients to the ICU. The application of the NISS or any of the mixed indices analyzed in the study (TRISS, NTRISS, BISS and BNISS) is feasible to predict mortality in ICU.

It is noteworthy that the characteristics of the studied sample corroborate the findings of other investigations regarding the mean age [8, 22] but differs in relation to the sex, main cause of trauma and severity of victims identified by anatomical indices. Studies published have identified falls as the second cause of trauma, preceded by traffic accidents, a higher prevalence of men as the main trauma victims [14, 15, 17] and calculated moderate to severe severity by the ISS and NISS indexes [5, 15, 17]. On the other hand, the mean and/or median values of the RTS and TRISS identified in this study corroborate findings from other international research [14, 17, 23].

The ICU admission rate (14.2%) of the sample was similar to that found in a study carried out in Tunisia in 2014 [17]. This frequency was considerably lower than the findings of other studies (30.0–81.0%) [14, 15, 24, 25]. It is known that patient outcome is the main focus of most studies that analyze trauma rates [14–17, 25], especially with the objective of identifying whether the investigated index was assertive in the survival probability. However, the number of fatal victims varies widely between studies, covering frequencies between 4.6% and 15% [14, 15, 17, 22], all superior to this research.

The results of this research showed that ISS and NISS had the best results in evaluating the performance of the indices for predicting patient admission to the ICU compared to the other indexes. Other investigations [17, 18] reinforce this finding, as they also identified good predictive capacity of anatomical indices for this condition.

Researchers analyzed 1,136 trauma victims treated at a hospital in Tunisia and found that the NISS (AUC 0.89) and ISS (AUC 0.91) had better results in predicting ICU admission in the sample than the Simplified Acute Physiology Scale II (SAPS II) (AUC 0.73) and RTS (0.58) [17]. An investigation that analyzed approximately 24,000 patients admitted to Trauma Centers in Quebec, Canada, showed that NISS (AUC 0.839) and ISS (AUC 0.843) were equivalent in discriminating patients admitted to the ICU; however, the NISS had better calibration for this outcome [18].

The findings of this study also showed that the NISS and the mixed indices showed excellent performance in predicting ICU patient mortality. A Spanish study analyzed the predictive capacity of TRISS for the mortality of trauma victims in the ICU and identified an AUC value of 0.887 for blunt trauma and 0.919 for penetrating trauma, constituting values close to those in this research [26]. A comparison of the TRISS (AUC 0.806) performance in relation to the Acute Physiology and Chronic Health Evaluation III—APACHE III (AUC 0.797) in predicting mortality in the ICU showed that the indices had the same accuracy, and the authors indicate the application of TRISS to evaluate this outcome since the index considers the characteristics of the trauma mechanism and the severity of the injuries [19].

Research carried out in Brazil identified that the performance of SAPS III (AUC 0.811), mREMS (AUC 0.802), RTS (AUC 0.747) and Rapid Emergency Medicine Score (REMS) (AUC 0.753) for predicting death in the ICU of surgical patients who suffered blunt trauma was similar and moderate, with no preferential indication of one of these indexes for use in the clinical practice of professionals [20].

The scarcity of studies in the literature did not enable a deeper discussion of the data in a comparative way with the scientific production, since most investigations in recent years have analyzed hospital mortality of trauma victims, and little has been addressed about the behavior of the indexes for other outcomes, such as admission and ICU mortality. However, there are recurrent attempts to improve the assertiveness of the indexes and adapt them to different realities, as evidenced by the high number of index proposals available in the literature [27].

Some limitations should be considered when applying the results of this study: (1) data were collected in a single trauma center in the city of São Paulo, Brazil; (2) the absence of arterial blood gas data upon patients’ admission to the emergency room did not enable calculating the BISS and BNISS indices to be performed in the sample as a whole, being restricted to patients hospitalized in the ICU; and (3) the reduced number of death cases in the ICU may have influenced the result of the performance of the indices in predicting mortality.

## Conclusions

The anatomical indices had a better predictive capacity for trauma patient admission to the ICU. The NISS and the mixed indexes had the best performances in relation to mortality. Applying the most assertive trauma index for ICU admission and mortality has the potential to help professionals in decision-making processes about resource allocation and strategies to improve the quality of patient care.

## Data Availability

The data sets used and analysed during the current study are available from the corresponding author on reasonable request.

## Abbreviations

AIS: Abbreviated injury scale
APACHE: Acute physiology and chronic health evaluation
AUC: Area under curve
BE: Base excess
BISS: Base-deficit injury severity score
BNISS: Base-deficit and new injury severity score
GCS: Glasgow coma scale
HR: Heart rate
ICU: Intensive care unit
ISS: Injury severity score
mREMS: Modified rapid emergency medicine score
NISS: New injury severity score
NPV: Negative predictive value
NTS: New trauma score
NTRISS: New trauma and injury severity score
PPV: Positive predictive value
REMS: Rapid emergency medicine score
ROC: Receiver operating characteristics curve
RTS: Revised trauma score
RR: Respiratory rate
SAPS: Simplified acute physiology scale
SBP: Systolic blood pressure
SpO_2_: Peripheral oxygen saturation
TRISS: Trauma and injury severity score
ΔBE: Base excess delta
